# Two Unexpected Temperature-Induced Supermolecular Isomers from Multi-Topic Carboxylic Acid: Hydrogen Bonding Layer or Helix Tube

**DOI:** 10.3390/molecules26226938

**Published:** 2021-11-17

**Authors:** Chunyang Li, Chunhong Tan, Juan Zhou, Yan-Yong Lin, Xiao-Feng Wang

**Affiliations:** 1 School of Chemistry and Chemical Engineering and Hunan Key Laboratory for the Design and Application of Actinide Complexes, University of South China, Hengyang 421001, China; lcy4657857@sina.com (C.L.); tanch2014@163.com (C.T.); 2 School of Mechanical Engineering, University of South China, Hengyang 421001, China; 3 School of Chemistry and Chemical Engineering, Guangzhou University, Guangzhou 510006, China

**Keywords:** tetra-carboxylic acid, temperature-induced, hydrogen bonding, supramolecular isomeric, crystal structure

## Abstract

Under ambient conditions or 160 °C, two supramolecular isomers, namely [(H_4_PTTA)(H_2_O)_2_(DMF)] and [(H_4_PTTA)(H_2_O)_3_]··Guest (**1-L** and **1-H**, H_4_PTTA = *N*-phenyl-*N*′-phenyl bicyclo[2.2.2]oct-7-ene-2,3,5,6-tetracarboxdiimide tetra-carboxylic acid, Guest = DMF and H_2_O), were obtained through the reaction of H_4_PTTA in a mixture of H_2_O and dimethylformamide. The single crystal structures reveal the temperature-dependent supramolecular isomerism derived from the torsion of semi-rigid of H_4_PTTA. The **1-L** prepared at room temperature is a hydrogen bond based achiral layer, while the hydrothermal synthesized **1-H** is isomer resulted in an H-bond-based chiral tubes-packed supramolecular framework.

## 1. Introduction

Crystal engineering, traced since 1955 and accelerated in the 1980’s by the independent efforts of Desiraju and other chemists, was further expanded as a fruitful research field encompassing various domains of chemistry, biology, pharmaceuticals and materials science [1,2,3,4,5]. In the last two decades, its development marks a shift from the analytical function of crystallography to the tuning of crystal structures, with the ultimate goal of the preparing of crystalline functional materials with promising properties [6,7,8,9,10]. In contrast to the hot topic branch of coordination polymers vial coordination bonds, the supramolecular synthesis for the preparation of new stable solid-state materials based on non-covalent interactions, which is the original research of crystal engineering, is still relatively obscure and should be strengthened.

One of the supramolecular fundamental principles is the consideration of intermolecular interactions between molecules in the same way as the covalent bonds connecting the atoms in the molecule [11]. Therefore, it is essential to understand the interactions of direct crystal packing and sustaining in order to assemble such supramolecular solids with the aid of different specific intermolecular contacts [12,13,14]. Besides aromatic stacking, hydrogen bonding frequently plays an overriding dominant factor in the rational design of supramolecular assemblies. For example, the prototypal organic networks of carboxylic acid systems are usually formed through the well-known $R_{2}^{2}$(8) eight-member carboxylic acid hydrogen bond dimers, which are considered as the concepts of supramolecular synthons [15]. Thus, the supramolecular synthon strategy has been widely applied to tailor co-crystals or supramolecular self-assemblies. Because of their strength and directionality, it is particularly useful when there are multiple functional groups capable of hydrogen bonding [16,17,18].

**Scheme 1:**
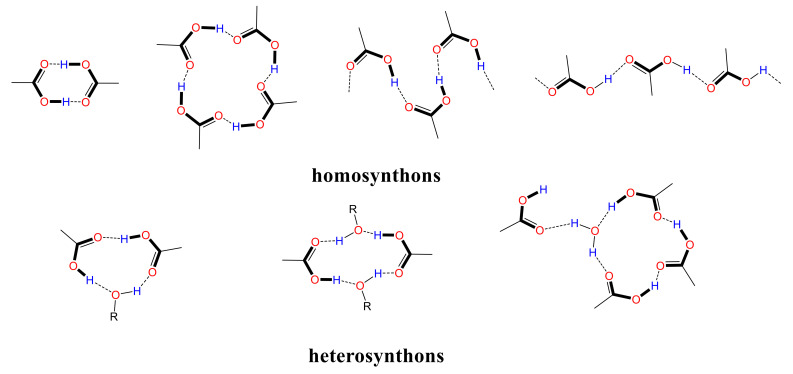
Supramolecular homosynthons (**top**) and heterosynthons (**bottom**).

Generally, the role of hydrogen-bond synthons is now well established in supramolecular crystal engineering, and it can be categorized into two types: the homosynthons from self-complementary half units, and the heterosynthons consisting of two or more components. The dimeric, tetramer or catemeric carboxyl···carboxyl homosynthons (Scheme 1 top) within the acid molecules dominate the interaction pattern for acid crystal structures [19,20,21]. Although the dimeric homosynthon acts as robust, it was demonstrated that it can be expanded or disrupted by the inclusion of water or alcohols (as both donor and acceptor), in order to be changed to the heterosynthons (one or two H_2_O symmetric inserting the dimmer, Scheme 1 bottom) involving -COOH and -OH, and consequently can bring up structural uncertainty [22,23,24].

The study of supramolecular isomerism is not only important in producing new materials but may also be helpful to fundamentally understanding the detail factors influencing the crystal growth process. Expected of co-crystals, the supramolecular isomerism, which comprises different structural networks derived from identical chemical compositions, is also attracting considerable investigative attentions. However, it is difficult and a great challenge to accurately predict or control the final supramolecular isomeric structures, owing to the subtle perturbation existence of thermodynamic and kinetic factors in the self-assembly process, such as different formation energy, building units’ variation or intermolecular interactions. Although some temperature-induced isomeric coordination polymers have been reported [25,26], the isomerism in a hydrogen bonding network is still rare [27,28]. Herein, we report two multitopic carboxylic acid-based isomers, where the solvent water molecules of solvation are incorporated, acting as the spacer characters to tune the homosynthon to heterosynthon, while being led to form the achiral layer at ambient or chiral tube at high temperature, respectively.

## 2. Experimental Section

### 2.1. General Materials and Methods

All the reagents and solvents were commercially available and used as received without further purification. The C, H and N elemental analyses were carried out on a Vario EL elemental analyzer (Elementar, Hesse, Germany). The Raman spectrum was recorded on a Thermo Scientific DXR 3 spectrometer using KBr pellets (Waltham, MA, USA). ^1^H liquid NMR spectrum was recorded with a Bruker AVANCE 300 spectrometer at 300.13 MHz (Billerica, MA, USA). Circular dichroism (CD) spectra were measured on a Jasco J-810 CD instrument (Tokyo, Japan).

*N*-phenyl-*N*’-phenyl bicyclo[2.2.2]oct-7-ene-2,3,5,6-tetracarboxdiimide tetracarboxylic acid (H_4_PTTA) was synthesized as described in the literature [29]: a mixture of 5-Aminoisophthalic acid (1.82 g, 10.05 mmol) and bicyclo[2.2.2]oct-7-ene-2,3,5,6-tetracarboxylic acid dianhydride (1.24 g, 5 mmol) in *N*,*N*’-Dimethylformamide (DMF, 20 mL) was refluxed for 12 h and then cooled down to room temperature. The yellow solution was poured into 500 mL deionized water and the pH was adjusted to 2 (with 5 M HCl) to form a white suspension. White powder was filtrated, washed twice with water and dried under vacuum at 50 °C to remove the solvent water. Raman (KBr) for H_4_PTTA: 3086 (m), 2960 (m), 1782 (m), 1609 (s), 1448 (w), 1391 (m), 1306 (w), 1238 (w), 1179 (m), 1089 (m), 1056 (w), 1003 (s), 940 (w), 837 (w), 799 (w), 735 (w), 699 (m), 634 (m), 588 (w), 382 (w), 216 (s), 112 (s) cm^−1^. ^1^H NMR (300 MHz, DMSO) δ (ppm): 8.48(t, J = 14, 2H, ph), 7.99 (d, J = 14.6, 4H, ph), 6.44~6.41 (m, 2H, -CH=), 3.58 (s, 2H, -CH), 3.50 (s, 4H, -CH). IR (KBr) for H_4_PTTA: 3460 (s, b), 1719 (s), 1609 (w), 1459 (m), 1383 (s), 1308 (w), 1274 (m), 1190 (s), 1118 (w), 1083 (w), 1048 (w), 948 (w), 793 (m), 758 (m), 682 (m), 622 (w), 527 (w), 497 (w) and 442 (w) cm^−1^.

#### 2.1.1. Preparation of [(H_4_PTTA)(H_2_O)_2_(DMF)] (**1-L**) 

To a solution of H_4_PTTA (0.162 g, 0.3 mmol) in DMF (5 mL) was added deionized water (100 mL), and then the pH value was adjusted to 2 using 2M HCl dropwise under constant stirring for 30 min. After filtered white precipitation, the clear filtrate was placed under ambient conditions; the colorless block-like crystals were isolated after slow evaporation of the solution in air for several weeks.

#### 2.1.2. Preparation of [(H_4_PTTA)(H_2_O)_3_]∙·Guest (**1-H**) 

H_4_PTTA (0.108 g, 0.2 mmol) was dissolved in a mixture of DMF (15 mL) and deionized water (5 mL) with constant stirring for 30 min. The clear solution was heated at 160 °C for 72 h under autogeneous pressure in a 25 mL sealed Teflon-lined stainless-steel vessel. After autoclave cooling to room temperature at a rate of 5 °C·h^−1^, colorless block crystals were isolated.

#### 2.1.3. X-ray Crystallographic Analysis

Single crystal data were collected on an Oxford CrysAlis CCD area detector diffractometer with graphite-monochromated Cu-Kα radiation (*λ* = 1.54184 Å) at 295(2) K. The structures were solved by direct methods and the subsequent difference Fourier synthesis was done using the SHELX-TL software suite [30]. Non-hydrogen atoms of the frameworks were refined anisotropically with hydrogen atoms generated as spheres riding the coordinates of their parent atoms. The solvent molecules in **1-H** were highly disordered and were impossible to refine using conventional discrete-atom models; thus, the contribution of solvent electron density was removed by the SQUEEZE routine in PLATON [31]. The crystallographic data and structure determination parameters are given in Table 1, and selected bond lengths and angles in Table 2. 

CCDC No. 2118337 and 2118346 contain the supplementary crystallographic data for the structures in this paper. These data can be obtained free of charge from the Cambridge Crystallographic Data Centre, CCDC, 12 Union Road, Cambridge CB2 1EZ, UK (Fax: +44-1223-336-033; or E-Mail: deposit@ccdc.cam.ac.uk, www.ccdc.cam.ac.uk, accessed on 11 September 2021).

## 3. Results and Discussion

### 3.1. Structural Description of [(H_4_PTTA)(H_2_O)_2_(DMF)] (***1-L***) 

**Figure 1:**
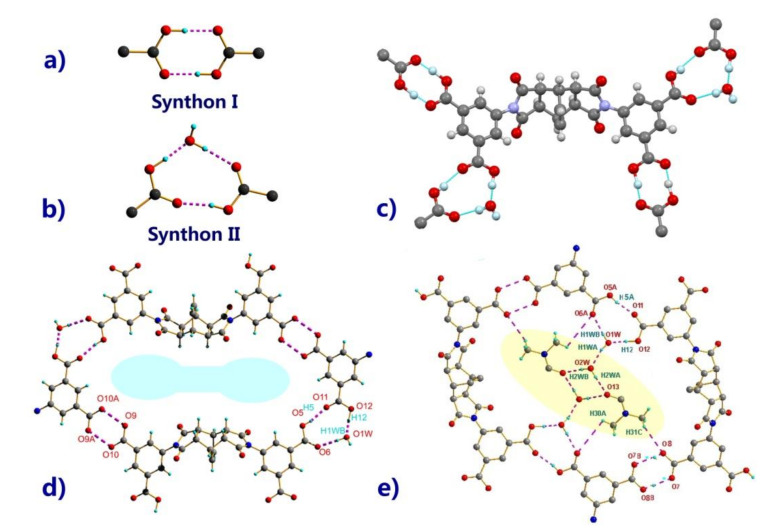
The homosynthon and heterosynthon (**a**,**b**); the H-bonds environment of one H_4_PTTA (**c**); two types of apertures: void (**d**) and fulfilled by lattice H_2_O and DMF (**e**) in **1-L**.

**Figure 2:**
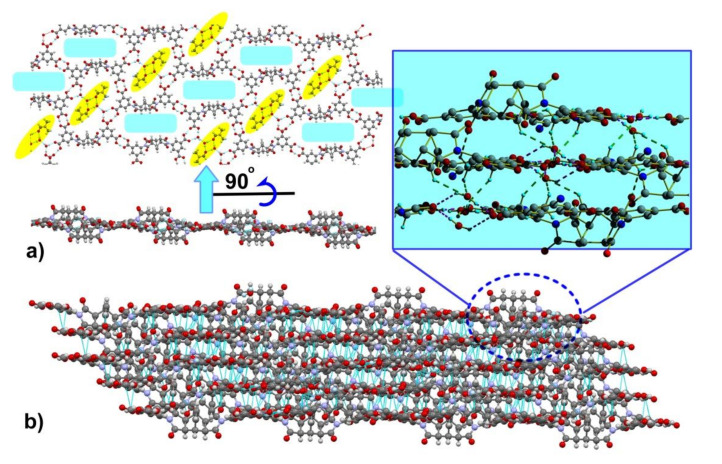
The top and side views of the two-dimensional hydrogen bond layer (**a**); the off-set packing of the two-dimensional hydrogen bonded sheets and interactions between adjacent layers (**b**) in **1-L**.

Solvates of H_4_PTTA from a DMF/water mixture solution under an ambient condition, namely **1-L**, gave high-quality crystals. As depicted in Figure 1, single crystal X-ray diffraction (SCXRD) shows that **1-L** crystallizes in triclinic system with centrosymmetric space group P-1. An asymmetric unit contains one crystallographically tetra-carboxylic acid H_4_PPTA molecule, two water molecules and one DMF molecule. The bicyclo[2.2.2]oct-2-ene unit in the H_4_PPTA is a boat-like conformation with the dihedral angle between pyrryl rings of 122.27°. The torsion angles between pyrryl and phenyl rings of isophthalic acid are 58.35° and 51.84°, respectively. The two phenyl rings from isophthalic acid moieties rings are non-coplanar and form dihedral angles of 23.72°, while both planes defined by the -COOH group are almost coplanar with the dihedral angle of 3.60°. Within the H_4_TPPA molecule, there are two types of strong hydrogen bond motifs: $R_{2}^{2}$(8) eight-member dimeric carboxylic homosynthon and one water inserted ten-member heterosynthon (Figure 1a). Each H_4_TPPA features two homosynthons and two heterosynthons. Two H_4_PTTAs combined with two isophthalic acid moieties from adjacent H_4_PTTA to form two different apertures: dumbell and oval, respectively (Figure 1d,e), while one was fulfilled with lattice H_2_O and DMF molecules. Besides these conventional hydrogen bonds, there are two unconventional C-H∙∙∙O hydrogen bonds between the -CH_3_ from the DMF and O atoms from two kinds of groups (carboxyl and aldehyde group with the C-H···O distances of 3.440 Å and 3.366 Å, respectively), which are not only a steric effect but also play a significant role in the formation of supramolecular assembly. These acid moieties are linked via two kinds of H-bond synthons to form a 2-D herringbone pattern layer along [100] direction (Figure 2a). Furthermore, the adjacent 2-D layer arrays stack in an off-set mode along the (100) direction, holding via weak C-H···O H-bonds between bicyclo[2.2.2]oct-2-ene unit and O atom from lattice water or pyrrolidine-2,5-dione segment to form a 3-D supramolecular structure. This suggests weak C-H···O interaction forces consolidate the whole supramolecular system.

### 3.2. Structural Description of of [(H_4_PTTA)(H_2_O)_3_]∙·Guest (***1-H***)

**Figure 3:**
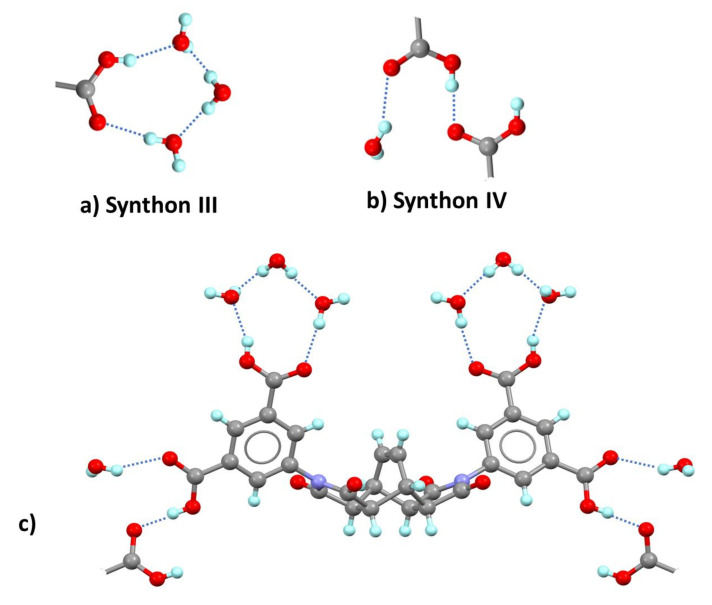
Two kinds of heterosynthons (**a**,**b**) and the H-bonds environment of one H_4_PTTA (**c**) in **1-H**.

**Figure 4:**
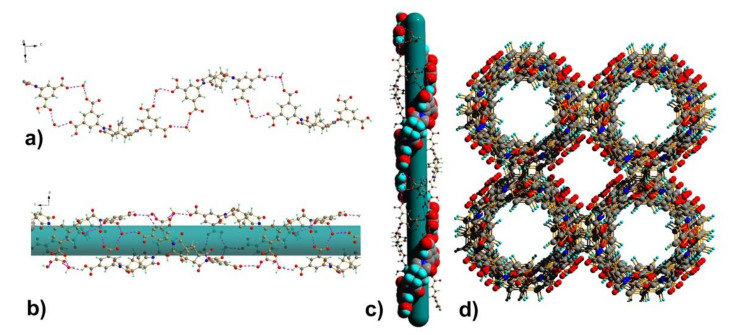
The mono-helix H-bond chain layer (**a**); the bi-helix chains interacted chiral H-bond tube (**b**,**c**); the packing of the hydrogen bonded tubes (**d**) in **1-H**.

Colorless block crystals can be obtained from DMF/water mixed solution at high temperature (160 °C), namely **1-H** with guest molecules of DMF and H_2_O. SCXRD shows that **1-H** crystallizes in a tetragonal system with chiral space group *P*4_3_22. The asymmetric unit contains a half of a crystallographically tetra-carboxylic acid H_4_PPTA molecule and one and a half water molecules. Similar to **1-L**, the bicyclo[2.2.2]oct-2-ene unit center of the H_4_PPTA molecule also adopted symmetric boat-like conformation, with a somewhat different dihedral angle between pyrryl rings of 123.68°. Within the H_4_TPPA molecule, the angles between phenyl rings of isophthalic acid segments and pyrryl rings are both 65.70°. It is worth noting that the angle of the phenyl ring plane of the isophthalic acid is obviously different from that in **1-L**, which is 39.25° larger than 23.72°. The two planes defined by the -COOH groups of the isophthalic acid segments are also non-coplanar with a dihedral angle of 4.93 or 16.59°, respectively. Each H_4_TPPA molecule holds eight hydrogen bonds featuring two kinds of H-bond motifs: three water inserted 11-member heterosynthons, and one water inserted 7-member heterosynthon (Figure 3a,b, respectively). Notably, in the 11-member heterosynthon, the H_2_O molecules feature different roles: one H_2_O acting as two H-bond donors (O1W-H···O, 2.884 Å), while another one acts as two H-bonds donors and two acceptors (O2W-H···O, 2.713, 2.784, 2.883 and 2.884 Å). Meanwhile, carboxylic groups act as H-bond donors and acceptors with a chelate model. The H_4_TPPA molecules are connected by these hydrogen bonds of two carboxylic acid groups: one is mode IV (Figure 3b), while another one is mode III (Figure 3a), in which the water molecule holds four hydrogen bonds as a bridge. In addition, the semi-rigid H_4_TPPA molecule has a C–N bond which can rotate with a certain angle, which hydrogen bonds combine together to construct a one-dimensional (1-D) infinite right-hand helix chain along the *b*-axis (see Figure 4a). Further, the adjacent 1-D chains array stack in an off-set mode along the *c*-axis to form right-hand helix tubes with a diameter of 18.22 Å, through intergral mode III and IV H-bond heterosynthons. The extended three-dimensional (3-D) homo-chiral supramolecular network with rhombic hydrophilic channels (fulfilled with guest molecules of DMF and H_2_O) is eventually constructed via interlayer hydrogen bonds involving the lattice water and resting carboxyl of H_4_TPPA tetra-acid (see Figure 4d).

### 3.3. Syntheses and Structural Comparison

**Scheme 2:**
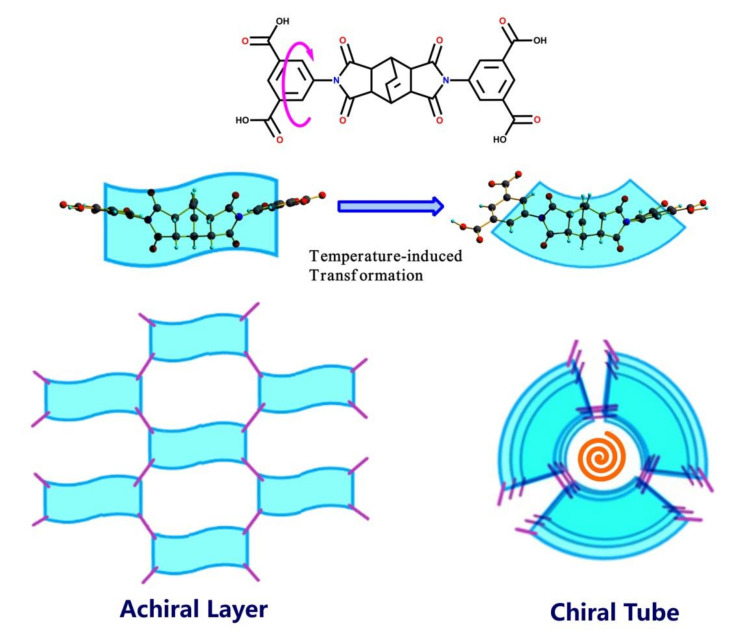
The supramolecular isomeric transformation derived from the ip segments torsion of semi-rigid tetra-acid ligands.

Two isomeric supramolecular nets, **1-L** and **1-H**, built by tetra-acid are synthesized from the same starting reactants under different temperatures. Owing to its semi-rigid rotation of the C–N bond, both phenyl rings of tetra-acid H_4_TPPA adopt dissimilar orientations with the changed torsion angles from 51.84° in **1-L** to 65.70° in **1-H**. The temperature-induced configuration transformation of H_4_TPPA indicates that the structural variation in the final isomeric formed by H-bond frameworks (Scheme 2) is influenced by the temperature applied during the synthesis. Thus, H-bonded wave-layer **1-L** is formed at the room temperature while the chiral H-bonded tube-like structure **1-H** is constructed at 160 °C under hydrothermal conditions. 

### 3.4. CD Spectroscopy

**Figure 5:**
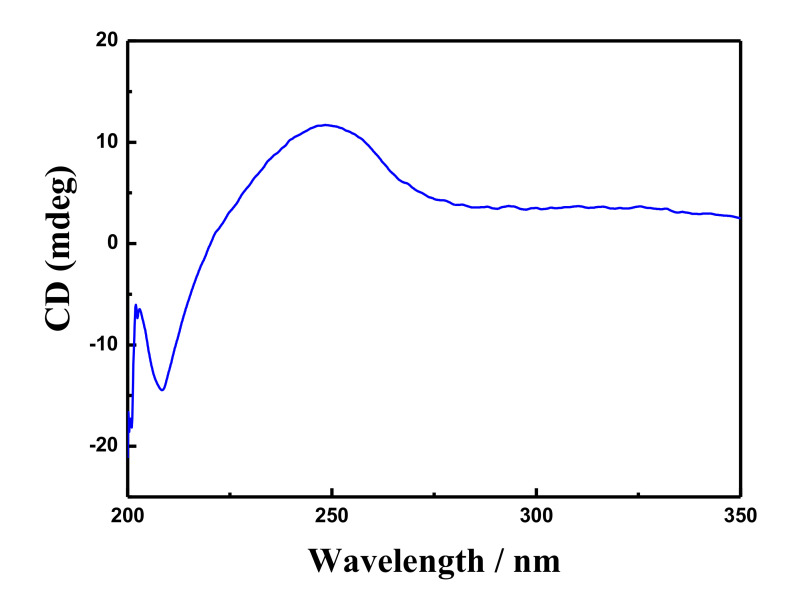
The solid-state circular dichroism (CD) spectra of stochastic of conglomerate **1-H**.

Analysis of the crystal structure suggests that **1-H** is a chiral supramolecular organic framework [32], which entails the self-assembly of homo-chiral (right-helix) tubes derived from the achiral semi-rigid tetra-acids being assembled via different hydrogen bond motifs. It has been confirmed in a kinetic model that an achiral artificial assembly system could spontaneously produce chirality [33]. However, without inducing the chiral chemical regents (as reactants or template), the coordinated or covalent enantiomers are commonly observed in the spontaneous resolution system [34,35,36,37]. Although **1-H** should contain two different enantiomer single crystals, unfortunately, the attempt to pick out and solve the left-helix structure of single-crystal X-ray crystallography failed. However, the circular dichroism (CD) measurement based on more than 50 randomly picked crystals from one-pot resulted in solid CD spectroscopy, showing that **1-H** explicitly exhibits Cotton effects at about λ = 215 and 255 nm, which confirms that the right- and left-helix enantiomers were spontaneously resoluted during the course of the crystallization in the one-pot hydrothermal reaction, as shown in Figure 5. The results indicate that more research into the rational syntheses strategy is required in order to figure out how to control the preparation of the homo-chiral H-bonded frameworks from spontaneous resolution.

## 4. Conclusions

In this work, we have successfully synthesized two supramolecular compounds, namely **1-L** and **1-H**, starting from the same precursors, the mixture of semi-rigid tetra-acid H_4_TPPA, H_2_O and DMF. It is interesting that both supramolecular compounds performed variable hydrogen bond isomers under different synthesis temperatures. The room temperature approach leads to the formation of **1-L**, which features the H-bond layer structure based on the general dimeric homosynthon and one H_2_O inserted dimmeric heterosynthon. **1-H** synthesized at 160 ºC under hydrothermal conditions, and results in the chiral H-bonded tube were based on two kinds of H-bond heterosynthons. Notably, the structural differences between two supramolecular isomers of **1-L** and **1-H** were derived from the rotation of the C-N bond of semi-rigid tetra-acid H_4_TPPA, which induced the different torsion of both isophthalic acid segments within H_4_TPPA.

## Figures and Tables

**Table 1:** The Crystallographic data for **1-L** and **1-H** ^a^.

	1-L	1-H
Empirical formula	C_31_H_29_N_3_O_1__5_	C_28_H_24_N_2_O_15_
Formula weight	683.59	628.49
Temperature	293(2)	296(2)
Crystal system	Triclinic	Tetragonal
Space group	*P*-1	*P*4_3_22
Unit cell dimensions		
a (Å)	7.0188(2)	11.0795(2)
b (Å)	13.4051(7)	11.0795(2)
c (Å)	17.4948(8)	35.0924(9)
α/° β/° γ/°	101.590(4) 98.090(3) 94.526(3)	90 90 90
Volume/Å^3^	1586.66(12)	4307.78(19)
*Z*	2	4
*D*_calc._/g·cm^−3^	1.431	0.969
*μ*(MoKα)/mm^−1^	0.994	0.080
*F*(000)	712	1304
*R*_int_	0.019	0.080
Reflections collected	9130	9844
Observed reflections [*I* > 2*σ*(*I*)]	4322	4446
Flack parameter	0	0.1(16)
Goodness-of-fit on *F*^2^	1.05	1.12
*R*_1_ [*I* > 2σ(*I*)]	0.0457	0.0461
*wR*_2_ (all data)	0.1226	0.1365
Largest diff. peak and hole/e.Å^−3^	1.08 and −0.47	0.29 and −0.28

^a^*R*_1_ = ∑||*F*_o_| − |*F*_c_||/∑|*F*_o_|·*wR*_2_ = [∑*w*(*F*_o_^2^ − *F*_c_^2^)^2^/∑*w*(*F*_o_^2^)^2^]^1/2^.

**Table 2:** Hydrogen bond parameters (Å, °) in the crystal structures of **1**-**L** and **1-H**.

1-L				
D-H···A	d(D-H)	d(H···A)	d(D···A)	<(DHA)
O1W-H1WA···O6 ^#1^	1.010	1.730	2.735(3)	172
O1W-H1WB···O2W	1.060	1.640	2.684(3)	167
O3-H3···O4 ^#2^	0.970	1.640	2.607(2)	178
O2W-H2WA···O13 ^#3^	0.890	1.880	2.768(4)	171
O2W-H2WB···O13 ^#4^	1.000	1.820	2.819(3)	173
O5-H5···O9 ^#5^	0.940	1.710	2.628(2)	166
O10-H10···O1W ^#3^	0.990	1.520	2.506(3)	173
O12-H12···O11 ^#6^	1.050	1.560	2.593(2)	168
C6-H6···O8 ^#1^	0.980	2.580	3.410(3)	143
C8-H8···O2 ^#7^	0.930	2.540	3.309(3)	140
C31-H31B···O2 ^#7^	0.960	2.510	3.366(4)	148
Symmetry codes: #1 = 2 − x, 2 − y, 1 − z; #2 = 1 − x, 2 − y, − z; #3 = − x, 1 − y, 1 − z; #4 = 1 + x, y, 1 + z; #5 = 2 + x, 1 + y, z; #6 = 1 − x, − y, 1 − z; #7 = − 1 + x, y, z.				
**1-H**				
**D-H···A**	**d(D-H)**	**d(H···A)**	**d(D···A)**	**<(DHA)**
O2W-H2WA···O1 ^#1^	0.85	2.11	2.883(2)	150
O2W-H2WB···O4 ^#2^	0.85	1.95	2.784(3)	167
O2-H2A···O2 ^#3^	0.86	1.6	2.440(2)	164
O3-H3···O2W	0.81	1.94	2.713(3)	160
O1W-H1WA···O2W	0.85	2.03	2.884(3)	177
C3-H3A···O3 ^#4^	0.98	2.46	3.076(6)	121
Symmetry codes: #1 = x, 2 − y, 1/2 − z; #2 = − 1 + y, 1 + x, 1/4 − z; #3 = x, 1 − y, 1/2 − z; #4 = − 1 + y, x, 1/4 − z.				

## Data Availability

Crystallographic data for (**1-L** and **1-H**) have been deposited with the Cambridge Crystallographic Data Center as supplemental publication numbers CCDC No. 2118337 and 2118346, respectively. Copies of the data can be obtained free of charge via http://www.ccdc.cam.ac.uk/data_request/cif, (accessed on 11 September 2021), or by emailing data_request@ccdc.cam.ac.uk, or by contacting The Cambridge Crystallographic Data Centre, 12, Union Road, Cambridge CB2 1EZ, UK.

## Sample Availability

Samples of the compounds are not available from the authors.
